# Targeting the gut microbiome in the management of sepsis-associated encephalopathy

**DOI:** 10.3389/fneur.2022.999035

**Published:** 2022-09-29

**Authors:** Brooke Barlow, Sameer Ponnaluri, Ashley Barlow, William Roth

**Affiliations:** ^1^Department of Pharmacy, Memorial Hermann The Woodlands Health System, Houston, TX, United States; ^2^Department of Neurology, University of Florida College of Medicine, Gainesville, FL, United States; ^3^Department of Pharmacy, MD Anderson Cancer Center, Houston, TX, United States

**Keywords:** gut microbiome, sepsis, sepsis-associated encephalopathy, T cell, fecal microbiota transplantation, Vagus nerve (VN) stimulation, probiotics

## Abstract

Brain injury resulting from sepsis, or sepsis-associated encephalopathy (SAE), occurs due to impaired end-organ perfusion, dysregulated inflammation affecting the central nervous system (CNS), blood-brain barrier (BBB) disruption, mitochondrial dysfunction, oxidative stress, accumulation of toxic neuropeptides and impaired toxin clearance secondary to sepsis-induced hepatic and renal dysfunction. The gut microbiome becomes pathologically altered in sepsis, which likely contributes to the pathogenesis of SAE. Herein, we review the literature detailing dysregulation of microbiota-gut-brain axis (MGBA) in SAE and highlight potential therapeutic strategies to modulate the gut microbiome to mitigate sepsis-induced brain injury.

## Sepsis-associated encephalopathy

Sepsis is one of the most common intensive care unit (ICU) syndromes and is one of the leading causes of death worldwide (1). Sepsis-induced injury to the central nervous system (CNS), known as sepsis-associated encephalopathy (SAE), remains a dangerously under recognized complication. SAE is defined as diffuse cerebral dysfunction occurring as a result of the systemic response to infection without clinical or laboratory evidence of brain infection or other etiologies of encephalopathy. Clinically, SAE manifests broadly from mild delirium and altered speech to coma (2). The diagnosis of SAE is a diagnosis of exclusion. Therefore, clinicians must first rule out other common causes of encephalopathy in patients with sepsis such as septic embolic infarcts, meningitis, intracranial hemorrhage, and other systemic complications of sepsis such as uremia. Several key drivers of SAE include systemic and central inflammation, permeabilization of the blood brain barrier (BBB), ischemia secondary to systemic vasodilation, mitochondrial dysfunction, concomitant metabolic derangements, accumulation of toxic neuropeptides (3).

Sepsis is the leading cause of ICU admissions, while estimates of SAE prevalence vary widely (anywhere from 9 to 71% of septic patients) due to inconsistent definitions of the condition high prevalence of comorbid conditions that also cause encephalopathy (4). One large retrospective study of 2,513 patients identified SAE in 53% of patients. Factors independently associated with SAE included acute renal failure, hypo- or hyperglycemia, hypercapnia, hypernatremia and infection with *S*. Aureus. Even mild SAE was associated with higher mortality (5). Another retrospective, single center study observed an incidence of SAE at 17.7% and found a significantly higher 28-day mortality (56.1 vs. 35.1% *p* = 0.013) and prolonged length of ICU stay in affected patients. The most prominent risk factors for SAE development included older age, previous cognitive impairment, organ impairment (kidney or liver dysfunction), and sepsis severity determined by the Acute Physiology and Chronic Health Evaluation (APACHE) II and Glasgow Coma Scale (GCS) (6). While definitions and prevalence estimates vary, SAE is consistently associated with greater disease severity and worse outcomes.

## The microbiota-gut-brain axis

The gut microbiome is the aggregate of all microorganisms living in the digestive tract of an organism. Over the past decade, research into the bidirectional influence of the gut microbiome, gut and central nervous system, known as the microbiota-gut-brain axis (MGBA), has accelerated rapidly. The MGBA is comprised of humoral, immune, endocrine, neural, and metabolic pathways by which the gut, gut microbiota and brain exert mutual influence. Many of these pathways become disrupted in sepsis and may contribute to the development of SAE.

In a healthy host, the gut microbiome maintains immune homeostasis and integrity of the gut barrier (7). The CNS influences gut-resident immune and enteric cells *via* neural and hormonal limbs of the MGBA, which in turn influence the gut microbiota (8). Gut microbiota influence the CNS by producing metabolites such as short chain fatty acids (SCFAs), that influence neural inflammation through modulation of neurotrophic factors and influence the phenotype and morphology of glial cells (9). Research has shown that the gut microbiome affects the CNS through modulation of the permeability of the blood brain barrier (10). Through examination of propionate interactions, a dietary substance produced by gut bacteria, the blood brain barrier was further demonstrated as another facet of the gut-brain axis (11). The CNS influences intestinal pH, motility and gastric emptying through differential activation of the autonomic and enteric nervous systems and through tuning of the hormonal stress response *via* the hypothalamo-pituitary-adrenal (HPA) axis. Additional metabolites produced by commensal microflora have been shown to mediate the activity of the Vagus nerve through endocrine and immune pathways (12, 13). Vagal activation by microorganisms influence serotonergic and GABAergic signaling in the primary afferent of the Vagus, the nucleus tractus solitarius (NTS), which projects to the parabrachial nucleus and amygdala, and alters behavior and cognition in mice (14). Gut microbiota, therefore, fortify pathways in physiologic conditions that become dysregulated in sepsis.

## The gut microbiome in sepsis

**Figure 1:**
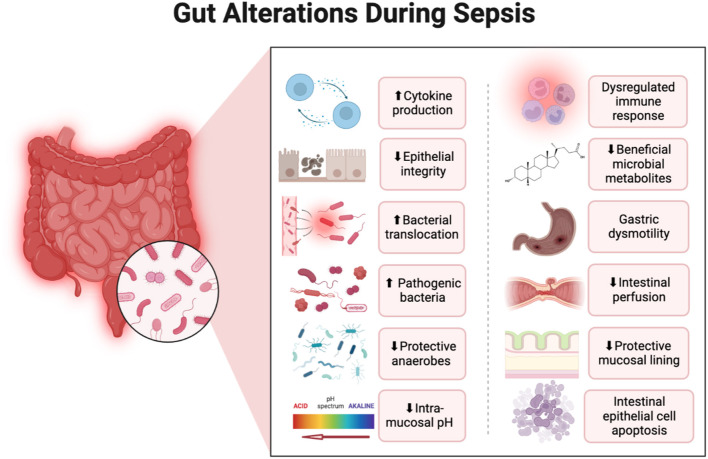
Alterations in the gastrointestinal system that occur during sepsis with a focus on changes to the gut microbiome. Created with Biorender.

**Figure 2:**
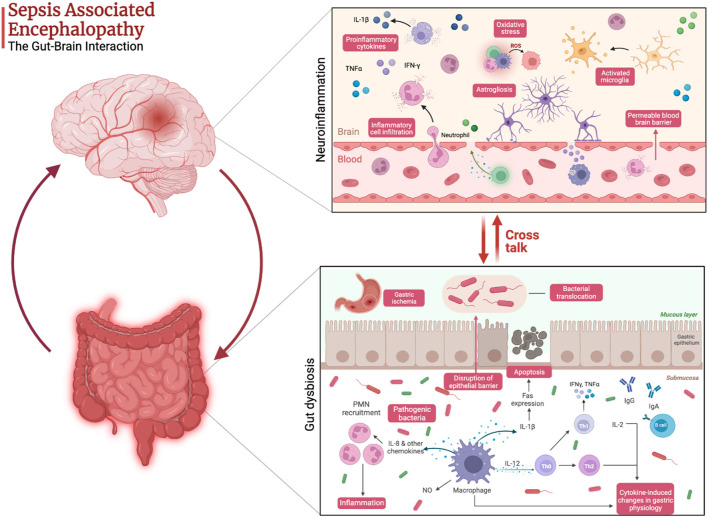
Pathophysiology of sepsis-associated encephalopathy highlighting the cross talk between gut dysbiosis and neuroinflammation. Created with Biorender.

Sepsis is associated with gut dysbiosis, a pathological disruption of gut microbial homeostasis (Figure 1). Pathogenic gut-resident organisms outgrow healthy commensal organisms. Patients with severe sepsis have reduced *Bifidobacterium* and *Lactobacillus* and increased colonization of pathogenic Staphylococcus and Pseudomonas spp (15). Krezalek et al. found that dysbiosis predicted sepsis-related mortality in their patient population (16). Sepsis induces intestinal wall permeability allowing bacterial antigens to interact with gut-resident immune cells and influence systemic immunity (17). Altered gastrointestinal barriers heighten the progression and severity of sepsis. In preclinical models, sepsis rapidly induces gut permeabilization (18). Endotoxin released from gut bacteria exacerbate systemic inflammation and promote organ damage (19). In addition to its role in the pathogenesis of sepsis, the gut microbiome also promotes the development of SAE. Endogenous gut microflora such as Bifidobacterium and Lactobacillus produce gamma-aminobutyric acid (GABA), the primary inhibitory neurotransmitter in the CNS, which can pathologically alter neural signaling. Concentration changes of these endogenous microflora observed in sepsis lead to the progression of altered mentation and have substantial impacts on CNS function. The reduction in SCFA production from gut microflora during sepsis increases inflammatory markers, endotoxins due to the inability to downregulate inflammation (20) (Figure 2).

## Impact of sepsis treatment on the gut microbiome

Therapies employed for sepsis and septic shock management also pathologically alter the constitution of the gut microbiome. Though crucial to infection management and mortality reduction in sepsis, antimicrobials have been shown to paradoxically increase bacterial infection-induced mortality secondary to perturbations in the intestinal microbiome (21, 22). Through indiscriminate depletion of commensal microbes and changes to the gut microenvironment, antibiotic overuse and misuse leads to a selective pressure that drives the generation and overgrowth of pathogenic bacteria such as *Clostridium difficile* and emergence of antibiotic resistance (23, 24). Antibiotics indirectly affect the gut microbiome through alteration of the host innate immune system. Antibiotic-associated depletion of gram-negative organisms reduces expression of toll-like receptors, signaling proteins and antimicrobial peptides (e.g., Reg3g) responsible for killing gram-positive organisms. Mice depleted of commensal gram-negative organisms demonstrated reduced clearance of vancomycin-resistant Enterococci (VRE) that was subsequently restored through enteral administration of lipopolysaccharide. Similar effects on gram-negative overgrowth occur with depletion of commensal gram-positive organisms (25).

Other medications commonly used in the critically ill septic patient such as corticosteroids, vasopressors, opioids, and proton pump inhibitors also promote gut dysbiosis. Catecholamines, such as norepinephrine, are the principal neurohormones released by the enteric nervous system during acute stress, such as in sepsis and septic shock. In the progression of sepsis to septic shock, profound changes in splanchnic circulation occur which reduce regional blood flow, reduce gastric motility, and impair gastric absorption. When impaired gastric perfusion is prolonged, risk of mesenteric ischemia ensues resulting in apoptosis and necrosis of the epithelial lining. Consequently, injured epithelial cells release damage associated molecular patterns (DAMPs) which further exacerbate the systemic inflammatory response and increase intestinal permeability (26). Exogenous catecholamine administration heightens the risk of perturbations in the gastrointestinal system. The potent vasoconstriction reduces splanchnic perfusion and increases gastric pH, signifying progression of gastric mucosal ischemia (27, 28). Epinephrine also exerts thermogenic effects, as it not only reduces gastric blood flow, but increases splanchnic oxygen consumption resulting in a splanchnic oxygen supply/demand mismatch (28). Several *in vitro* studies have also linked direct gut bacteria exposure to norepinephrine with an increase bacterial growth, *de novo* synthesis of virulence factors, and bacterial translocation due to increased gut permeability (29–32). *In vitro* models suggest norepinephrine may serve as an endogenous siderophore, interfering with iron sequestration and increasing free iron availability to enhance growth of gram-negative bacteria (33).

Corticosteroids play a crucial role in the management of septic shock to restore the alterations in the HPA axis and correct the absolute or relative adrenal insufficiency that can contribute to prolonged, persistent shock. In addition, corticosteroids are critical in the host response to infection. Though their anti-inflammatory and immunomodulatory effects are well-known, elevation in glucocorticoid concentrations may also reduce gut microbiome diversity (34). Rodents treated with a single dose of dexamethasone developed increases in the number of pathogenic anaerobic and coliform bacteria (35). Glucocorticoid administration in mice suppresses colonic mucin expression which reduces intestinal integrity and enhances the risk of colonic inflammation (36).

Opioid analgesics remain a mainstay therapy for pain management in critically ill patients, including those with sepsis and septic shock (37). However, evidence has recently emerged implicating opioids as important risk factors in the development of sepsis and septic shock by promoting gut dysbiosis. Opioid exposure in animal models of sepsis have shown opioids induce direct mucosal injury, compromising the protective gut barrier and increasing the risk of bacterial translocation (38, 39). Morphine administration in mice with sepsis altered the expression of key regulatory cytokines resulting in propagation of gram-positive bacteria and enhanced virulence of gram-negative organisms such as *Pseudomonas aeruginosa* (40, 41). Chronic opioid use has also been shown to alter the composition of gut microflora and confer increased risk of infection. In patients with cirrhosis, chronic opioid use was associated with an increased risk of gut dysbiosis, endotoxemia, and all-cause readmission rates compared to no opioid use (42). Opioids also directly impair adaptive and innate immune responses, further heightening the risk of infection (43).

Proton pump inhibitors (PPI), frequently employed for stress ulcer prophylaxis in ICU patients, substantially alter gut microbiome diversity and consequently increase pathogenic bacterial expression. PPI use is associated with a 65% increased risk of developing *C. difficile* infections and a 2.5-fold increased risk of infection from other enteric bacteria such as *Salmonella* and *Campylobacter* (44, 45). Stool sampling of 211 patients with chronic PPI use demonstrated an abundance of oral microflora in the gut, reduction in healthy microflora with an increased growth of pathogenic bacteria including *Enterococcus, Streptococcus, Staphylococcus* and *Escherichia coli*, compared to non-PPI users (46).

Recognition of the potential risk of gut dysbiosis with the pharmacotherapeutics employed in the management of sepsis and sepsis shock by clinicians may beneficially alter practices.

## Potential therapeutic interventions for SAE targeting the gut microbiome

While various therapeutic strategies to treat or mitigate the risk of SAE in critically ill patients have been tested, no evidence-based therapy exists to date. Focus on the gut microbiome and its contribution to SAE has become an area of growing interest. Limited evidence is now available for the use of prebiotics, probiotics, fecal microbiota transplantation, and Vagus nerve stimulation for the treatment and prevention of SAE. Several investigational therapies demonstrating neuroprotective benefits, including palmitoylethanolamide, KAT5 inhibitors, capsase-1 inhibitors and butyrate supplementation for SAE remain in the therapeutic pipeline (20, 47, 48). Dexmedetomidine exhibits anti-inflammatory effects *in vitro* and its potential therapeutic benefit in the setting of SAE being actively investigated in the Adjunctive Sedation With Dexmedetomidine for the Prevention of Severe Inflammation and Septic Encephalopathy (ADVISE) trial (49).

### Probiotics

Probiotics are live microorganisms that lack inherent virulence properties and confer a health benefit to the host (50). While significant variations exist in preparations, practitioners most commonly prescribe *Lactobacillus-* and *Bifidobacterium-*containing probiotics. Probiotics mitigate brain dysfunction in SAE through modulation of the gut microbiome in conjunction with suppression of inflammatory cytokine expression. Probiotics are hypothesized to create a favorable gut microenvironment by decreasing luminal pH, reducing gut permeability, improving mucosal integrity and decreasing bacterial translocation (50, 51). In addition, probiotics suppress overgrowth of pathogenic bacteria, promote IgA production, and exhibit immunomodulatory properties to reduce intestinal inflammation (52). In contrast to probiotics which are live organisms, prebiotics are non-digestible nutrients that promote commensal bacterial growth. Synbiotics, the combination of prebiotics and probiotics, allow these agents to work synergistically to regulate the gut microbiome.

Changes in gut microbiota after probiotic supplementation with *Lactobacillus* or *Bifidobacterium* has been shown in human and animal models to reduce psychological stress, anxiety, and improve cognition and memory in association with suppression of proinflammatory cytokine expression (53–55). Recognition of these potential neuroprotective benefits of probiotics has paved way for their investigation in the management of SAE. Li and colleagues assessed the neuroprotective effects of probiotic administration in a SAE mouse model. Mice with SAE administered an intragastric probiotic containing *Clostridium butyricum* (Cb) for 1 month exhibited improved cognitive effects, a significant reduction in neuroinflammation and increased brain-derived neurotrophic factor (BDNF) (56).

Despite the proposed advantages of probiotics, randomized controlled trials evaluating their impact on sepsis or sepsis related complications in critically ill patients are inconclusive (57–59). In a meta-analysis evaluating 2972 critically ill patients, probiotics reduced the rate of infections by 20% (risk ratio 0.80, CI 0.68–0.95) but had no impact on mortality or ICU length of stay. However, in more recent randomized controlled trials, these effects were not demonstrated and, further, some immunocompromised patients developed lactobacillus bacteremia. While the risk of bacteremia has been associated with the probiotic strain containing *Lactobacillus rhamnous* GG, the potential for other probiotic strains to induce a similar effect cannot be dismissed (57, 60). Additional evidence from a systematic review and meta-analysis evaluating the safety of probiotics suggests an association between probiotics and the risk of sepsis, fungemia, and gastric ischemia, with critically ill patients at the highest risk (61). Cases of fungemia, some resulting in death, were reported in critically ill patients receiving probiotics containing *Saccharomyces boulardii*, whereas bacteremia was more frequently associated with Lactobacillus strains (61). Probiotic use is also limited by the variability and inconsistencies in product formulations. Probiotics inhibit the growth and virulence of pathogenic bacteria through production of proteins called bacteriocins which have varied antimicrobial properties. Probiotics deficient in bacteriocin gene expression are less effective in inhibiting pathogenic bacterial growth (62). Defining the optional preparation, dose, and administration frequency, efficacy and safety profile of probiotic administration in critically ill patients with SAE remains to be fully elucidated.

### Fecal microbiota transplantation

While probiotics focus on supplementing single or small groups of bacterial species, fecal microbiota transplantation (FMT) provides a novel strategy to replace the entire intestinal ecosystem. FMT takes a complete intestinal ecosystem from a healthy individual, including a diverse array of microbial species in varied proportions along with the necessary substrates and metabolites responsible for immunomodulation (e.g., short chain fatty acids, D-lactate) and transfers this flora into the diseased recipient to restore the gut microbiome in its entirety (63). This strategy offers the most comprehensive strategy to correct the aberrations in microbial diversity secondary to sepsis. FMT restores the dynamic balance of bacterial species and their essential metabolites that exhibit beneficial anti-inflammatory effects to reduce the harmful neuroinflammatory mediators that contribute to SAE (64). FMT has demonstrated a potential neuroprotective benefit in the prevention or treatment of hepatic encephalopathy, epilepsy, Parkinson's disease and neuropsychiatric conditions such as depression, autism spectrum disorder, and anxiety (65–67). The additional anti-inflammatory and autoimmune properties of FMT have expanded its utility in the use of multiple sclerosis and systemic inflammatory response syndrome (SIRS) (67). In a case series of critically ill patients with SIRS unresponsive to antibiotics (*n* = 5), administration of FMT resulted in rapid defervescence, blood culture clearance and other measures of clinical improvement. One patient demonstrated a rapid reduction in inflammatory cytokines and normalization of Th1/Th2 and Th1/Th17 ratios (68).

Evidence for use of FMT in critically ill patients is limited and only animal models exist for use in SAE. In critical illness, case reports on the use of FMT to address gut dysbiosis demonstrated a consistent reduction in inflammatory mediators and normalization of Th1/Th2 and Th1/Th17 ratios (69). Xi and colleagues conducted a study of SAE-induced mice in order to investigate the key mediators that describe the relationship between intestinal flora disturbances and the development SAE. The mice were randomly assigned to receive FMT or a sham procedure and subsequently followed to assess changes in cognition, changes in the gut microbiome using 16s rRNA sequencing, and changes in systemic and neuroinflammatory markers using blood samples and hippocampal tissue, respectively. FMT restored neurocognitive deficits associated with SAE which correlated with the improvement in lesions and inflammatory markers found within hippocampal tissues. The neurocognitive benefits correlated with a significant reduction in inflammatory cytokines IL-1β, IL-6, and TNF- α, apoptosis, and concentrations of the excitatory neurotransmitter glutamate. Inhibition of intestinal exosome section of IL-1β reversed neurologic deficits associated with SAE, a hypothesis further supported by the reemergence behavioral changes upon exogenous recombinant IL-1β administration (70).

Li and colleagues discovered that among four different methods of gut microbiota modulation in septic rats (prebiotics, probiotics, FMT and synbiotics), FMT was found to be the most efficacious in restoring intestinal microbial diversity and exhibited a neuroprotective effect in SAE (71). FMT induced a significant reversal of LPS-induced delta and theta activity excess, low voltage, absence of reactivity, triphasic waves and periodic discharges on EEG in addition to a reduction in neuroinflammatory cytokines, IL-6, TNF-α, and IL-1β derived from cerebral cortex (71). The authors hypothesized that reconstitution of microbial diversity with increased presence of commensal, beneficial bacterial species such as *Bifidobacterium, Lactobacillus*, and *Faecalibacterium* and reduction in noxious bacteria contributed to the neuroprotective benefits of FMT therapy.

Adverse events associated with FMT include rare inflammatory, infectious and procedural complications. Cases of gram-negative bacteremia and subsequent death secondary to aspiration during the procedure or due to bacterial translocation have been reported with FMT (72, 73). Patients with underlying immunocompromised conditions or those with an acute immunocompromising illness are proposed to be at heightened risk of bacteremia and septic complications (63). Implementation of FMT into clinical practice presents numerous practical challenges given the specialized equipment, training and regulatory requirements. FMT is a multistep, time-sensitive process and includes appropriate patient identification, or selection, preparation of fecal material, fecal delivery/administration and post-FMT monitoring. Delivery of fecal transplant can occur *via* the enteral route, colonoscopy or an enema, with route depending on institution and patient specific factors (69). In its capacity to transplant microbiota and metabolites, it represents a promising avenue for further implementation in patients with SAE.

### Vagus nerve stimulation

Vagus nerve stimulation represents another novel therapeutic approach to suppressing neuroinflammation. Classified as a mixed nerve, the Vagus nerve plays an important role in both efferent and afferent signal transmission to regulate the function of various internal organs, glands, and involuntary muscles throughout the body. The Vagus plays a critical, yet underrecognized role in the innate inflammatory reflex. Located in the jugular ganglia, the inflammatory reflex arc begins with afferent Vagus nerve activation by peripheral cytokine release which transmits information to neurons in the nucleus tractus solitarius (NTS) located in the medulla oblongata. Evidence suggests efferent Vagus nerve endings located in the superior mesenteric and celiac ganglia release acetylcholine to activate the noradrenergic post-ganglionic splenic nerve which induces release of acetylcholine (ACh) from T cholinergic lymphocytes, which in turn activate the α7 subunit-containing nicotinic acetylcholine receptor to suppress synthesis and release of inflammatory cytokines from splenic macrophages. This reflex arc is activated in conditions of systemic inflammatory condition such as sepsis (74, 75). In recognition of the role of the Vagus nerve in modulating inflammation, interest in the potential benefits of this neuroimmune pathway as a potential therapy in sepsis and other inflammatory diseases has grown (76).

Vagus nerve stimulation (VNS) is a form of neuromodulation that involves manual or electrical activation of the vagus nerve. Modulation of the cholinergic pathway through vagal nerve stimulation (VNS) in mice has been shown to reduce LPS-induced TNF-α secretion and a reduction in intestinal inflammation. In patients undergoing colorectal surgery, abdominal VNS reduced serum levels of LPS-induced IL-8 and IL-6 suggesting beneficial anti-inflammatory effects (77).

In the setting of SAE, Sun and colleagues investigated the role of the Vagus nerve in the management of SAE-induced mice. Twenty mice were randomly assigned to one of four groups, 1) control, 2) lipopolysaccharide (LPS), 3) FMT plus LPS, and 4) LPS plus vagotomy and FMT. Changes in gut microflora, brain dysfunction and cerebral cortex concentrations of TNF-α, IL-1β, IL-6 and IL-10 levels were measured to assess neuroinflammation. Compared to LPS alone, both groups treated with FMT had near complete restitution of intestinal microbial diversity, with a reduction in pathogenic bacteria and an increase in commensal organisms like that of controls. Treatment with FMT in septic mice improved cognition, spatial learning, reduced neuroinflammatory markers present in the hippocampus and diminished microglial activation, all of which were diminished in mice treated with FMT plus vagotomy. FMT also alleviated EEG evidence of brain dysfunction which was reversed by vagotomy. Collectively, this study presents novel insight on the critical importance of the Vagus nerve in the MGBA and further represents an area of research for its potential benefit in the treatment of SAE.

## Application to clinical practice

The gut microbiome alterations that occur in sepsis may contribute to the development of SAE and, in turn, worse outcomes for affected patients. While no clinical trials investigating microbial modulation strategies have yet been undertaken, preclinical models show promise for implementation of interventions employed for other disease processes, such as probiotics, FMT and VNS. These interventions appear to mitigate the myriad ways in which sepsis causes and potentiates brain injury: by halting disruption of neurotransmitter and bioactive neuropeptide concentrations, altering Vagal neural signaling to attenuate neuroinflammation and modify neural circuits important for behavior and cognition, and hampering toxic microbial antigen exposure at the microbiota-gut interface. Time to treatment initiation and treatment duration for SAE remain ill defined. However, given the alterations in gut microbiota occur in the early in the course of sepsis, prompt and early initiation is likely to be more impactful in altering the disease course.

## Conclusions

Leveraging the growing understanding of the influence of the MGBA in the pathogenesis of SAE through precision-based microbial modulation strategies has significant promise for the treatment and prevention of this condition. Emerging data supports further steps toward employment of these strategies in clinical trials.

## Author contributions

All authors contributed equally to the development of this manuscript.

## Conflict of interest

The authors declare that the research was conducted in the absence of any commercial or financial relationships that could be construed as a potential conflict of interest.

## Publisher's note

All claims expressed in this article are solely those of the authors and do not necessarily represent those of their affiliated organizations, or those of the publisher, the editors and the reviewers. Any product that may be evaluated in this article, or claim that may be made by its manufacturer, is not guaranteed or endorsed by the publisher.
